# Comparison of the genetic relationship between nine Cephalopod species based on cluster analysis of karyotype evolutionary distance

**DOI:** 10.3897/CompCytogen.v11i3.12752

**Published:** 2017-07-25

**Authors:** Jin-hai Wang, Xiao-dong Zheng

**Affiliations:** 1; 2 Laboratory of Shellfish Genetics and Breeding, Fisheries College, Ocean University of China, Qingdao 266003, China; 3 Institute of Evolution and Marine Biodiversity, Ocean University of China, Qingdao 266003, China

**Keywords:** octopods, cytogenetics, chromosome, genetic relationship, evolutionary distance

## Abstract

Karyotype analysis was carried out on gill cells of three species of octopods using a conventional air-drying method. The karyotype results showed that all the three species have the same diploid chromosome number, 2n=60, but with different karyograms as 2n=38M+6SM+8ST+8T, FN (fundamental number)=104 (*Cistopus chinensis* Zheng et al., 2012), 2n=42M+6SM+4ST+8T, FN=108 (*Octopus minor* (Sasaki, 1920)) and 2n=32M+16SM+12T, FN=108 (*Amphioctopus fangsiao* (d’Orbigny, 1839–1841)). These findings were combined with data from earlier studies to infer the genetic relationships between nine species via cluster analysis using the karyotype evolutionary distance (*D_e_*) and resemblance-near coefficient (*λ*). The resulting tree revealed a clear distinction between different families and orders which was substantially consistent with molecular phylogenies. The smallest intraspecific evolutionary distance (*D_e_*=0.2013, 0.2399) and largest resemblance-near coefficient (*λ*=0.8184, 0.7871) appeared between *O. minor* and *C. chinensis*, and *Sepia esculenta* Hoyle, 1885 and *S. lycidas* Gray, 1849, respectively, indicating that these species have the closest relationship. The largest evolutionary gap appeared between species with complicated karyotypes and species with simple karyotypes. Cluster analysis of *D_e_* and *λ* provides information to supplement traditional taxonomy and molecular systematics, and it would serve as an important auxiliary for routine phylogenetic study.

## Introduction

Cephalopoda is an old and evolutionarily successful molluscan group with a worldwide distribution (Jazayeri et al. 2011, Adachi et al. 2014). It includes several species that are precious marine resources but are difficult to manage due to their short life span and sensitivity to environmental conditions (Emery et al. 2016). Extant cephalopods are divided into two subclasses: Nautiloidea and Coleoidea. Members of Coleoidea are main catch targets and are common in fish markets (Lu 2000). Approximately 134 cephalopod species (Lu et al. 2012), including commercially important marine species such as *Octopus minor* (Sasaki, 1920), *Amphioctopus fangsiao* (d’Orbigny, 1839–1841), *Cistopus chinensis* Zheng et al., 2012 and *Sepia esculenta* Hoyle, 1885, are found in Chinese waters. According to the China fishery statistical yearbook (Zhao 2016), cephalopod landings totalled nearly 0.7 million tonnes in 2015, with an increase of 3.42% over the previous year. Because of the high economic benefits surrounding octopods, many intensive studies have investigated their population genetics (Zheng et al. 2009, Meriam et al. 2015, Gao et al. 2016), behaviour (Meisel et al. 2013, Polese et al. 2015, Levy et al. 2015, Richter et al. 2016), neurology (Nixon and Young 2004, Zarrella et al. 2015), and reproductive biology (Wada et al. 2006, Ebisawa et al. 2011, Wang et al. 2015b). However, while significant genetic knowledge is required for effective breeding and aquaculture of octopods, modern cytogenetic studies of these species are scarce.

Karyotype analysis is the foundation of cytogenetic studies, playing an important role in understanding the origin and evolution of organisms by studying the variation in the number or structure of their chromosomes (Chung et al. 2012). Despite the importance of understanding the role of chromosomes in cephalopod evolution, chromosome research in these species is poorly developed because of their huge diploid chromosomes and the lack of good split phases. The most reliable karyotype information comes from Gao and Natsukari (1990), who studied two octopods *O. ocellatus* Gray, 1849 (*A. fangsiao*) (Jereb 2014) and *O. vulgaris* Cuvier, 1797, two sepiids (*S. esculenta* and *S. lycidas* Gray, 1849) and three loliginids (*Heterololigo bleekeri* Natsukari, 1984, *Sepioteuthis lessoniana* Blainville, 1824 and *Photololigo edulis* (Hoyle, 1885)) (Table 1). Earlier studies led by Inaba and Vitturi reported the chromosome number of *O. vulgaris*, *O. minor* and *S. officinalis* Linnaeus, 1758 (Inaba 1959, Vitturi et al. 1982), but included no detailed karyotype description. In the last three decades, only a scant few publications have been focused on cephalopod karyotype research. Bonnaud et al. (2004) reported the *Nautilus macromphalus* Sowerby, 1849 karyotype, with 52 chromosomes, and other studies revealed the chromosome number of Gulf cuttlefish (*S. arabica* Massy, 1916 and *S. pharaonis* Ehrenberg, 1831) via examination of the blood cells (Papan et al. 2010, Jazayeri et al. 2011). However, the findings of these follow-up studies remain uncertain since they lacked ideal division phases and basic chromosome parameters. Similarly, recent karyotype analyses of *S. esculenta* and *O. areolatus* de Haan, 1839–1841 (*A. fangsiao*) (Jereb 2014) have been revealing but were not sufficiently thorough (Wang et al. 2011, Adachi et al. 2014). In general, to obtain satisfactory split phases, embryos are better; however, this method is severely constrained by the availability and accessibility of material during the cephalopod breeding season. In addition, the use of germ cells is also restricted by season, and chromosomes are short during this period, which is not conducive to routine karyotype analysis (Zhang et al. 2007). Gills provide an alternative source for karyotyping which is convenient, fast, and not subject to seasonal restrictions; however, due to the slow metabolism of adults, there is little cell division in this tissue. Together, these factors act to limit cephalopod chromosome studies.

**Table 1.** Basic karyotype information of nine species of cephalopods.

**Species**	**Origin**		**Karyotype**			**References**
	**Locations**	**Materials**	**2n**	**FN**	**Formulas**	
*O. minor*	Weihai, Shandong Province, China	gills	60	108	42M+6SM+4ST+8T	This study
*O. vulgaris*	Nagasaki, Japan	embryos	60	76	14M+2SM+8ST+36T	Gao and Natsukari (1990)
*A. fangsiao*	Qingdao, Shandong Province, China	gills	60	108	32M+16SM+12T	This study
*C .chinensis*	Ningde, Fujian Province, China	gills	60	104	38M+6SM+8ST+8T	This study
*S. lycidas*	Ohmura, Nagasaki, Japan	wild eggs	92	172	66M+14SM+10ST+2T	Gao and Natsukari (1990)
*S. esculenta*	Shimabara, Nagasaki, Japan	wild eggs	92	164	48M+24SM+14ST+6T	Gao and Natsukari (1990)
*S. lessoniana*	Nomozaki, Nagasaki, Japan	wild eggs	92	156	54M+10SM+24ST+4T	Gao and Natsukari (1990)
*P. edulis*	Nagasaki, Japan	embryos	92	160	50M+18SM+16ST+8T	Gao and Natsukari (1990)
*H. bleekeri*	Nagasaki, Japan	embryos	92	166	54M+20SM+18ST	Gao and Natsukari (1990)

Karyotype evolutionary distance has been used as an important parameter in studying the classification and evolution of animals. In this approach, the distance of karyotype evolution (*D_e_*) and resemblance-near coefficients (*λ*) are estimated from the karyotype data by mathematical statistics based on the principles of numerical taxonomy and similar analysis theory, and these parameters accurately reflect the interspecific or intraspecific relationship at the cytological level. While the classification and genetic relationships of cephalopods is a continuing topic of interest and has been addressed using molecular systematics tools, such as mitochondrial DNA (Cheng et al. 2013, Zhang et al. 2015), without reaching a consensus, evidence from chromosome morphology is still seldom used to analyse the relationships and evolution of cephalopod taxa (Thiriot-Quiévreux 2003). Determining the genetic relationships between species based on cellular characteristics would be an effective supplement to traditional taxonomy and molecular systematics, and would serve as an important auxiliary means of routine analysis.

Here, we use a cytogenetic approach to study the genetic relationships of cephalopods at the chromosome level. We used gills to obtain good metaphase mitotic plates, and then calculated the *D_e_* and *λ* in order to construct a cluster analysis diagram among nine species cephalopods. These findings enrich our knowledge of cephalopod chromosome structure and provide a new and important index for cephalopod taxonomic classification and the determination of genetic relationships at the cytological level.

## Material and methods

### Specimens

We obtained ten live *O. minor* specimens from the Rongcheng coastal waters of the Bohai Sea (37°13'N, 122°33'E), Shandong Province, China, and ten specimens of *A. fangsiao* were from the Qingdao coastal waters of the Yellow Sea (36°06'N, 120°32'E), Shandong Province, China. Another ten *C. chinensis* was transported to laboratory in plastic bags with oxygenation, at a low temperature, from the Ningde coastal waters of the East Sea (27°18'N, 119°32'E), Fujian Province, China. All individuals were about 40g and were identified based on morphological characteristics.

### Chromosome preparation

Chromosome preparation followed the method of Gao and Natsukari (1990) with some modifications. Briefly, the octopods were cultured in a 0.01% colchicine solution for 12h. In keeping with the PETA protocols, the gills were rapidly immersed in a 0.075M KCl solution for 1 hour, then the conventional air-drying method was applied. After indoor drying, the slides were stained with a 5% Giemsa solution for 10 min following the protocol used by Okumura et al. (1995). They were then observed under a light microscope with an oil lens (Leica MC170 HD, Germany).

### Construction of karyo-idiograms

Microphotographs of the chromosomes were used for karyotype analysis with Image-Pro Plus 6.0 (Wang et al. 2015a). Chromosomes were extracted from the original images, with homologous chromosome pairing and sorting based on visual observation. Chromosomes were classified adhering to Levan et al. (1964), and the length index was calculated according to Kobayashi (1986). Using these criteria, we automatically generated a schematic showing the long and short arms with different colours based on the measured values. A notch to represent the centromere was added to each chromosome using SmoothDraw. Finally, homologous chromosomes were arranged below the diagrams with Image-Pro Plus 6.0.

### Cluster analysis

We used the chromosome relative length as karyotype parameter of nine species (three from this study) for the analysis of evolutionary relationships (Table 1). *D_e_* and *λ* values were calculated with preliminary statistical analysis according the proposed criterion (for details, see Supplemental formulae). Further data analysis through SPSS 19.0 and Microsoft Excel 2007, the *D_e_* data matrix was then incorporated into a MEGA5.0 (Tamura et al. 2011) genetic distance operation document (.meg), and the karyotype evolution distance cluster tree was constructed.

## Results

### Karyotype analysis

**Figure 1.**
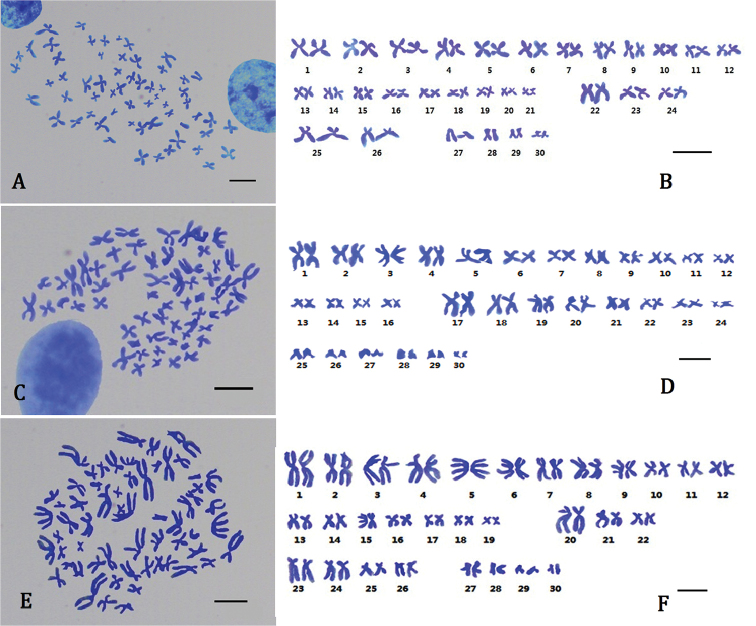
Photomicrographs of somatic diploid metaphase plates and karyotypes from three species of octopod gills. **A** The metaphase plate of *O. minor* **B** Karyogram of *O. minor* from (**A**) showing the karyotype composition: 42 metacentric (#1–#21), 6 submetacentric (#22–#24), 4 subtelocentric (#25–#26), and 8 telocentric (#27–#30) chromosomes **C** The metaphase plate of *A. fangsiao* **D** Karyogram of *A. fangsiao* from (**C**) showing the karyotype composition: 32 metacentric (#1–#16), 16 submetacentric (#17–#24), and 12 telocentric (#25–#30) chromosomes **E** The metaphase plate of *C. chinensis* **F** Karyogram of *C. chinensis* from (**E**) showing the karyotype composition: 38 metacentric (#1–#19), 6 submetacentric (#20–#22), 8 subtelocentric (#23–#26), and 8 telocentric (#27–#30) chromosomes. Scale bar 5 μm.

Karyological analysis of Giemsa-stained chromosomes was successfully obtained from at least seven well- divided metaphase plates from the studied populations of *O. minor*, *A. fangsiao* and *C. chinensis* (Fig. 1), and measurements of the chromosomes are shown in Table 2. All three octopods had a diploid chromosome number of 2n=60. The *O. minor* karyotype was 2n=42M+6SM+4ST+8T (FN=108), composed of 21 pairs of metacentric (1st-21st), 3 pairs of submetacentric (22nd-24th), 2 pairs of subtelocentric (25th-26th), and 4 pairs of telocentric (27th-30th) chromosomes. The relative length of each chromosome ranged from 1.15 to 4.99. In all metaphases we observed, the arm ratio (AR) of the 22^nd^ pair chromosomes was greater than or equal to 1.70, making it a submetacentric chromosome pair according the centromeric index (CI). The *A. fangsiao* karyotype was 2n=32M+16SM+12T (FN=108), consisting of 16 pairs of metacentric (1st-16th), 8 pairs of submetacentric (17th-24th), and 6 pairs of telocentric (25th-30th) chromosomes. The relative length of each chromosome ranged from 0.90 to 6.88. Finally, the *C. chinensis* karyotype was 2n=38M+6SM+8ST+8T (FN=104), consisting of 19 pairs of metacentric (1st-19th), 3 pairs of submetacentric (20th-22nd), 4 pairs of subtelocentric (23rd-26th), and 4 pairs of telocentric (27th-30th) chromosomes. The relative length of each chromosome ranged from 1.56 to 8.28. From the karyotype formulas, we found that *A. fangsiao* had no subtelocentric chromosomes, while *O. minor* and *C. chinensis* had quite close karyotypes, with differences only in the (sub)metacentric chromosomes. It is obvious that the metacentric and submetacentric chromosomes account for most of the chromosomes (>73.3%) (Fig. 4), indicating that they are derived with a higher classification status.

**Table 2.** Comparison of karyotype parameters obtained among *O. minor*, *A. fangsiao* and *C. chinensis*. (SA, short arm relative length; LA, long arm relative length; AR, arm ratio=LA/SA; CI, centromeric index=SA/(SA+LA) ×100; M, metacentric, 1.0 < AR < 1.7; SM, submetacentric, 1.7 < AR < 3.0; ST, subtelocentric, 3.0 < AR < 7.0; T, telocentric, 7.0 < AR. Values as mean ± SE)

Chromosome no.	*Octopus minor* (42M+6SM+4ST+8T)						*Amphioctopus fangsiao* (32M+16SM+12T)						*Cistopus chinensis* (38M+6SM+8ST+8T)					
	SA	LA	SA+LA	AR	CI	Type	SA	LA	SA+LA	AR	CI	Type	SA	LA	SA+LA	AR	CI	Type
1	2.43	2.54	4.97±0.17	1.05±0.15	48.89	M	3.33	3.55	6.88±0.18	1.06±0.08	48.46	M	4.13	4.15	8.28±0.05	1.00±0.10	49.88	M
2	2.28	2.49	4.77±0.11	1.09±0.12	47.80	M	2.99	3.46	6.45±0.70	1.16±0.12	46.36	M	3.64	3.75	7.39±0.11	1.03±0.02	49.26	M
3	2.22	2.51	4.73±0.05	1.13±0.08	46.93	M	3.04	3.15	6.19±0.47	1.03±0.02	49.17	M	3.60	3.78	7.38±0.07	1.05±0.02	48.78	M
4	2.30	2.43	4.73±0.13	1.06±0.12	48.63	M	2.86	2.87	5.73±0.19	1.00±0.11	49.91	M	3.15	3.44	6.59±0.12	1.09±0.03	47.80	M
5	2.36	2.36	4.72±0.06	1.00±0.13	50.00	M	2.53	2.96	5.49±0.07	1.17±0.17	46.01	M	3.33	3.35	6.68±0.09	1.01±0.11	49.85	M
6	2.28	2.36	4.64±0.01	1.04±0.05	49.14	M	1.94	2.12	4.06±0.20	1.09±0.04	47.78	M	3.14	3.18	6.32±0.15	1.01±0.04	49.68	M
7	2.21	2.38	4.59±0.05	1.08±0.01	48.15	M	1.97	2.09	4.06±0.10	1.06±0.01	48.52	M	2.97	3.19	6.16±0.04	1.07±0.01	48.21	M
8	2.19	2.40	4.59±0.11	1.10±0.14	47.71	M	1.66	2.18	3.84±0.21	1.31±0.01	43.23	M	2.83	2.98	5.81±0.20	1.05±0.07	48.71	M
9	2.16	2.38	4.54±0.05	1.12±0.02	47.58	M	1.82	1.91	3.73±0.22	1.05±0.02	48.79	M	2.42	2.48	4.91±0.17	1.02±0.11	49.29	M
10	2.13	2.15	4.28±0.12	1.01±0.10	49.78	M	1.63	2.07	3.70±0.06	1.27±0.01	44.05	M	2.29	2.44	4.73±0.06	1.07±0.06	48.41	M
11	2.12	2.16	4.28±0.03	1.01±0.07	49.53	M	1.55	1.95	3.50±0.31	1.26±0.08	44.29	M	2.28	2.45	4.73±0.15	1.07±0.08	48.20	M
12	2.03	2.17	4.20±0.14	1.07±0.13	48.33	M	1.68	1.76	3.44±0.11	1.05±0.04	48.84	M	2.24	2.27	4.51±0.11	1.01±0.03	49.67	M
13	1.92	2.04	3.96±0.07	1.06±0.09	48.48	M	1.42	1.79	3.21±0.27	1.26±0.21	44.24	M	1.99	2.31	4.40±0.07	1.16±0.01	45.23	M
14	1.91	1.98	3.89±0.15	1.04±0.14	49.10	M	1.56	1.64	3.20±0.04	1.05±0.13	48.75	M	1.96	2.27	4.23±0.10	1.16±0.03	46.34	M
15	1.88	1.98	3.86±0.09	1.05±0.10	48.70	M	1.47	1.69	3.16±0.32	1.15±0.32	46.52	M	2.03	2.10	4.13±0.16	1.03±0.12	49.15	M
16	1.36	1.90	3.26±0.11	1.40±0.13	41.72	M	1.32	1.69	3.01±0.05	1.28±0.13	43.85	M	1.75	1.99	3.74±0.05	1.14±0.07	46.79	M
17	1.42	1.54	2.96±0.12	1.08±0.18	47.97	M	2.48	4.39	6.87±0.13	1.77±0.28	36.10	SM	1.58	1.96	3.54±0.03	1.24±0.08	44.63	M
18	1.28	1.29	2.57±0.05	1.01±0.10	49.81	M	2.37	4.30	6.67±0.07	1.81±0.14	35.53	SM	1.55	1.55	3.10±0.02	1.00±0.04	50.00	M
19	1.24	1.27	2.51±0.13	1.02±0.11	49.40	M	1.92	3.59	5.51±0.24	1.87±0.29	34.85	SM	1.02	1.23	2.25±0.04	1.21±0.06	45.33	M
20	0.93	1.19	2.12±0.08	1.28±0.10	43.87	M	2.02	3.46	5.48±0.09	1.71±0.12	36.86	SM	1.52	4.53	6.05±0.08	2.98±0.06	25.12	SM
21	0.95	0.96	1.91±0.10	1.01±0.05	49.74	M	1.53	2.66	4.19±0.26	1.74±0.20	36.52	SM	1.23	2.46	3.69±0.16	2.00±0.20	33.33	SM
22	1.85	3.14	4.99±0.05	1.70±0.05	37.07	SM	1.12	2.50	3.62±0.18	2.23±0.11	30.94	SM	1.02	2.42	3.44±0.08	2.37±0.08	29.65	SM
23	1.01	2.87	3.88±0.10	2.84±0.11	35.19	SM	1.05	2.53	3.58±0.19	2.41±0.01	29.33	SM	1.15	4.63	5.78±0.09	4.03±0.04	19.90	ST
24	0.97	2.90	3.87±0.09	2.99±0.02	25.06	SM	0.74	2.13	2.87±0.03	2.88±0.30	25.78	SM	1.10	3.89	4.99±0.03	3.54±0.01	22.04	ST
25	1.03	3.52	4.55±0.13	3.42±0.13	22.64	ST	0.32	2.86	3.18±0.66	8.90±0.13	10.10	T	0.55	3.35	3.90±0.16	6.09±0.13	14.10	ST
26	0.83	3.17	4.00±0.01	3.82±0.04	20.75	ST	0.22	2.88	3.10±0.40	12.90±0.32	7.20	T	0.73	3.15	3.88±0.10	4.32±0.13	18.81	ST
27	-	3.02	3.02±0.01	∞	-	T	-	2.71	2.71±0.42	∞	-	T	-	2.71	2.71±0.02	∞	-	T
28	-	3.02	3.02±0.05	∞	-	T	-	2.54	2.54±0.16	∞	-	T	-	2.74	2.74±0.06	∞	-	T
29	-	1.77	1.77±0.01	∞	-	T	-	1.90	1.90±0.03	∞	-	T	-	1.69	1.69±0.07	∞	-	T
30	-	1.15	1.15±0.07	∞	-	T	-	0.90	0.90±0.03	∞	-	T	-	1.56	1.56±0.10	∞	-	T

We compared the relative chromosome length of the nine species of cephalopods and plotted a detailed chromosome distribution diagram to show the number and proportion of the different types of chromosome in the different species (Fig. 4). *S. lycidas* had the highest proportion of metacentric chromosomes (M, up to 71.7%), while the lowest appeared in *A. fangsiao* (below 23.5%). They correspondingly had the lowest and highest proportion of telocentric chromosomes (T, 2.2% and 60.0%). The four chromosome types (M, SM, ST and T) made up 56.9%, 16.6%, 14.5% and 12.0%, respectively, of the total chromosomes in the cephalopod karyotypes. Metacentric and submetacentric chromosomes were the major components of the karyotypes of Octopodiformes and Decapodiformes, accounting for 65.0% and 77.8% of the chromosomes, respectively. In almost all nine species, M was the largest proportion of chromosome types (with a minimum of 52.2%), followed by SM, while the other two types had variable proportions. The only exception was *O. vulgaris*, in which the highest proportion was T chromosomes (up to 60.0%), followed by M (23.3%), ST (13.3%), and SM (3.4%). These differences suggest that *O. vulgaris* may have experienced comparatively large chromosomal rearrangements, such as translocations or inversions, during its evolution.

### Construction of karyo-idiograms

**Figure 2.**
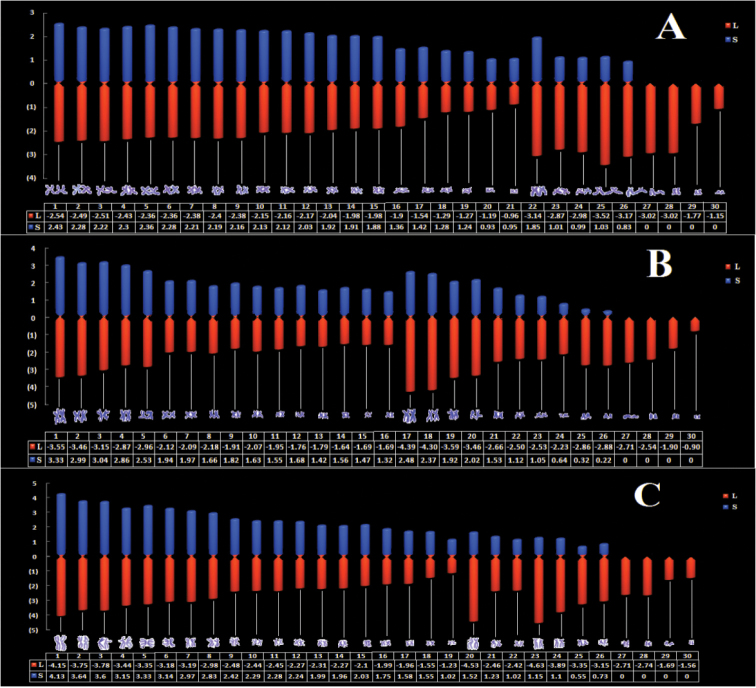
A novel display method of karyo-idiograms. Three octopods are shown: **A** *O. minor* **B** *A. fangsiao* **C** *C. chinensis*. The blue columns are the short arms and the red columns are long arms. Nicks mark the centromeres.

We developed a novel method to create normative karyo-idiograms of the three species based on the karyotype parameters (Fig. 2). The diagrams vividly and intuitively show the basic characteristics of each chromosome. The zero point in the diagram is the location of the centromere, and the chromosomes are arranged according to their type and size.

### Genetic relationship analysis

Karyotypes vary greatly between species, with greater karyotype evolutionary distance (*D_e_*) and smaller resemblance-near coefficients (*λ*) between distantly related species. Likewise, the karyotype evolutionary distance within a family is generally smaller than that between different families. To make an integrative analysis of the genetic relationships, the *D_e_* and *λ* values of the nine cephalopods were calculated (Table 3). *D_e_* measures ranged from 0.2013 to 1.3323, with an average of 0.6742. The largest *D_e_* was between *A. fangsiao* and *H. bleekeri* (Keferstein, 1866), whereas the smallest distance was between *O. minor* and *C. chinensis*. Correspondingly, the largest estimate for *λ* was between *O. minor* and *C. chinensis*, whereas the smallest estimate was between *A. fangsiao* and *H. bleekeri*. Overall, the *λ* values ranged from 0.2640 to 0.8184, with an average of 0.5283. In the Decapodiformes (Sepioidea and Teuthoidea), *S. esculenta* and *S. lycidas* had the closest relationship, with the smallest *D_e_* (0.2399).

**Table 3.** The karyotype evolutionary distance and resemblance-near coefficient among nine species of cephalopods.

Species	*O. minor*	*O. vulgaris*	*A. fangsiao*	*C. chinensis*	*S. lycidas*	*S. esculenta*	*S. lessoniana*	*P. edulis*	*H. bleekeri*
***O. minor***		0.5894	0.7594	0.8184	0.5244	0.3747	0.4401	0.4839	0.2742
***O. vulgaris***	0.5291		0.5495	0.5540	0.4183	0.3765	0.4725	0.4392	0.3057
***A. fangsiao***	0.2760	0.6000		0.7976	0.5423	0.5075	0.4515	0.4963	0.2640
***C. chinensis***	0.2013	0.5912	0.2262		0.5343	0.4744	0.5467	0.3846	0.3663
***S. lycidas***	0.6460	0.8722	0.6122	0.6271		0.7871	0.6328	0.6297	0.5990
***S. esculenta***	0.9809	0.9782	0.6776	0.7471	0.2399		0.5809	0.5594	0.6051
***S. lessoniana***	0.8214	0.7494	0.7960	0.6034	0.4570	0.5431		0.5280	0.3650
***P. edulis***	0.7265	0.8230	0.7011	0.9550	0.4620	0.5822	0.5904		0.5984
***H. bleekeri***	1.2954	1.1845	1.3323	1.0101	0.5120	0.5030	0.6940	0.5140	

Note: The evolutionary distance is in the left lower quadrant, and the resemblance-near coefficient is in the right upper quadrant.

**Figure 3.**
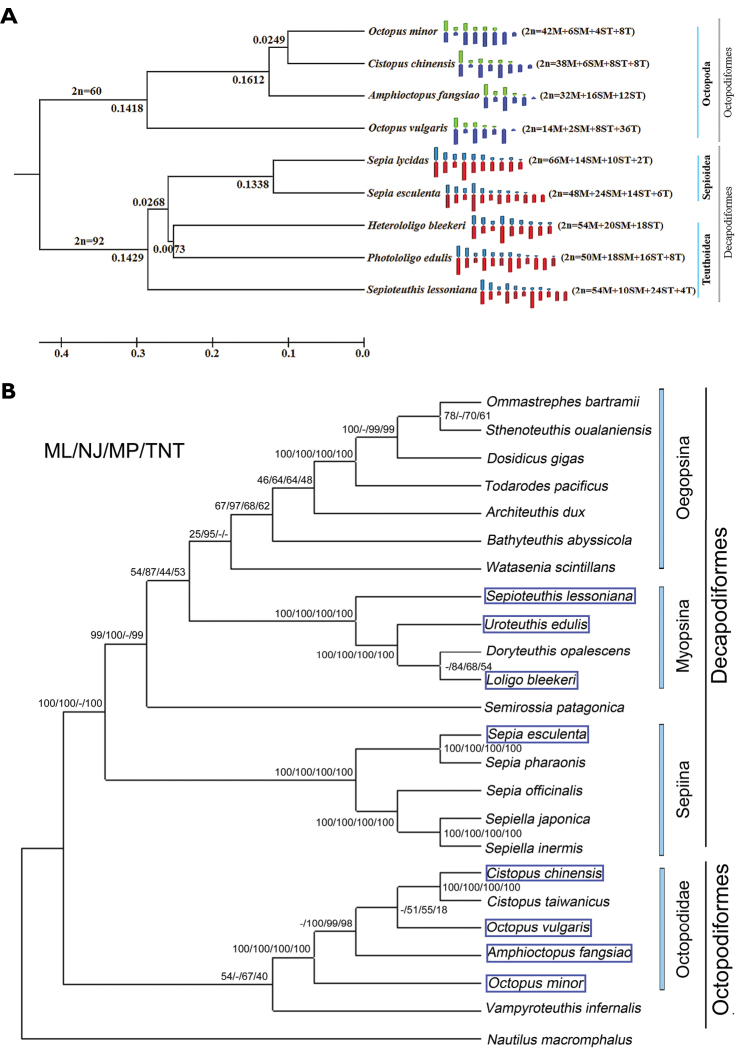
**A** Relationships between chromosome number and UPGMA clustering of nine species of cephalopods by evolutionary distance with simplified karyo-idiogram and karyotype formulas. Chromosome numbers and *D_e_* values are shown on the corresponding branches **B** Phylogenetic relationships among the cephalopods based on mitochondrial DNA sequences including the nine species of this study (Cheng et al. 2013).

In order to shed further light on phylogenetic divergence within the clades Octopoda, Sepiida and Teuthida, a cluster analysis was applied (Fig. 3A). The results showed clear distinctions between the different families and orders which were not quite concordant with the phylogenetic analysis at the molecular level. Decapodiformes and Octopodiformes (Octopoda) were definitely classified as two major clades. The four species in the order Octopoda clustered together as clade I, with *D_e_*=0.1418, while species from the orders Sepiida and Teuthida form a second clade, with *D_e_*=0.1429. Within clade I, *O. minor* and *C. chinensis* clustered as a monophyletic group with the smallest *D_e_* (0.0249), indicating the closest relationship, while *A. fangsiao* appeared as a sister group with *D_e_*=0.1612; *O. vulgaris* formed a sister to the three other octopod species. In clade II, formed by five species of the Decapodiformes, *S. esculenta* and *S. lycidas* formed one monophyletic group and *H. bleekeri* and *P. edulis* formed a second, sister monophyletic group, with *D_e_*=0.1338 and 0.0073, respectively, while *S. lessoniana* was as a sister to the two monophyletic groups.

## Discussion

In previous reports, germ cells, blood cells, and embryos (Inaba 1959, Gao and Natsukari 1990, Papan et al. 2010, Jazayeri et al. 2011, Adachi et al. 2014) have been used in cephalopod karyological studies, but this is the first study to use gill cells as a source of chromosomes, from which we were able to obtain positive metaphase plates.

The chromosome number of the three species in the present study was 2n=60, which is consistent with previous karyotype studies of octopods (Gao and Natsukari 1990, Adachi et al. 2014). However, in the present study, the *A. fangsiao* karyotype (32SM+16SM+12T) had twelve telocentric chromosomes, which disagrees with Gao and Natsukari 1990 (32M+28SM) and Adachi et al. 2014 (48M+8M/SM+4SM), who contend that this species has only M and SM chromosomes. Furthermore, the karyotype formula we found for this species was different from previous reports, which may be due to differences in sampling and preparation methods causing chromosome polymorphism, which is common in shellfish (Wang et al. 2015a). Arslan and Zima (2015) also emphasized that a cytotype may include several populations with different karyotypes despite having the same diploid number of chromosomes. Similarly, chromosomal diversity and differentiation has been confirmed in creepers (Manthey et al. 2015). In addition, the present study revealed a new karyotype of *O. minor* which was clearly different from results of Inaba (1959), who reported a diploid chromosome number of 56 in spermatogonia and primary spermatocytes. However, the lack of dependable metaphase division and detailed chromosomal parameters leads us to doubt the earlier result and favour the current study. Zhang et al.(2007) also pointed out that chromosomes obtained from sperm cells were too small to observe.

**Figure 4.**
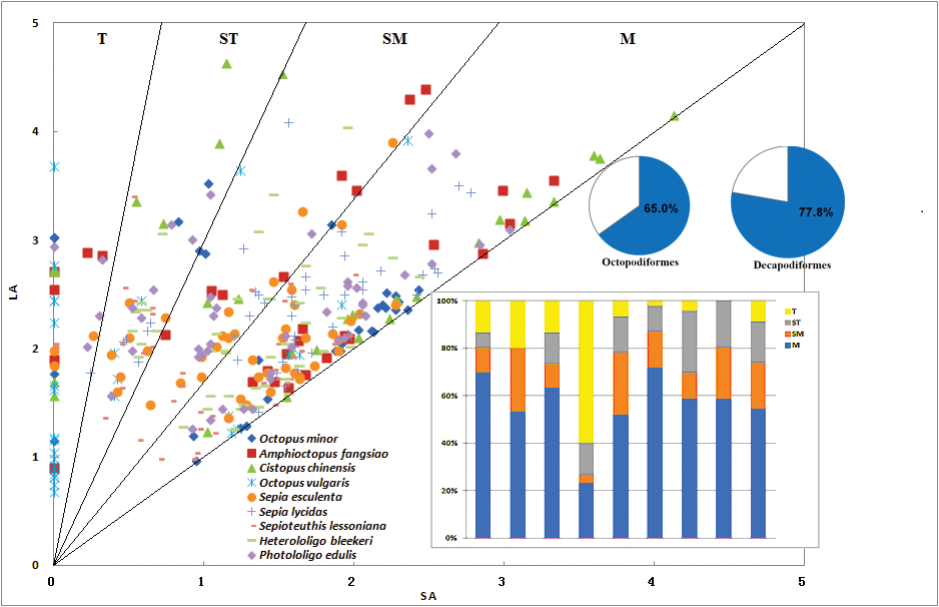
Chromosome distribution diagram of nine species of cephalopods. The slopes of the four lines are 1, 1.7, 3 and 7, dividing the diagram into four zones which represent four types of chromosome. SA, short arm relative length; LA, long arm relative length; M, metacentric; SM, submetacentric; ST, subtelocentric; T, telocentric. The blue area of pie charts and bar charts means M+SM and M, respectively.

Despite the three octopods having the same number of chromosomes, the karyotypes were remarkably different from each other. Compared with *O. minor* and *C. chinensis*, *A. fangsiao* had a specialized karyotype without ST, while the former two had almost the same karyotype, with only slight differences in M and ST (Fig. 4). Based on the findings reported in the present study and in Gao and Natsukari (1990), the three species of order Teuthida and the two species of the order Sepiida should have a higher classification status than the four species of the order Octopoda because they have a significantly greater diploid chromosome number (92 vs. 60), which is consistent with the results of the cluster analysis (Fig. 3). Together, M and SM were the main components of karyotype, suggesting that the cephalopods have a higher classification status. Similar observations have been made in bivalve shellfish, where karyotypes with a majority of metacentric-submetacentric chromosomes were characteristic of most bivalve species (Thiriot-Quiévreux 2002). Interestingly, *S. lycidas* and *A. fangsiao* contained the highest and lowest proportion of M and the lowest and highest proportion of T chromosomes.

Earlier analyses of cephalopod genetic relationships mainly concentrated on phylogenetic reconstruction via specific rDNA sequences (Bonnaud et al. 2004, Cheng et al. 2013, Zhang et al. 2015). Comparison of the karyological characters of the nine species of cephalopods for which data are available at the cytological level with a cluster analysis using karyotype evolutionary distance yielded substantial agreement with the phylogeny based on mitochondrial genes (Fig. 3B). Within clade II, the two species of the order Sepiida and three species of the order Teuthoidea formed different groups, which is concordant with the reported of Cheng et al. (2013), while larger divergence appeared in the order Octopoda. *C. chinensis* and *C. taiwanicus* Liao & Lu, 2009 formed a monophyletic group, with *O. vulgaris* and *A. fangsiao* appeared as sisters to the above group, while *O. minor* was a sister to the combined group. A subsequent publication validated these phylogenetic relationships based on an analysis of the complete mitochondrial genome (Zhang et al. 2015). In both cases, there was a closer genetic relationship between *C. chinensis* and *O. vulgaris* than with *A. fangsiao* and *O. minor*. However, the present study found the closest relationship between *C. chinensis* and *O. minor*, which formed a clade with the smallest *D_e_* (Table 3 and Fig. 3A). The taxonomic status of *O. minor* has been in dispute, with a recent study assigning it to the genus *Callistoctopus* Taki, 1964 based on CO1 and CO3 phylogenetic analyses (Kaneko et al. 2011). From the present analysis, we concluded that chromosome number and type played a leading role in clustering, since some species grouped together as a clade based on chromosome number, while others clustered separately into different branches based on karyotype similarity. For example, *C. chinensis* and *O. minor* readily clustered together based on the similarity of their karyotype, while *O. vulgaris* had a special karyotype which deviated from the other three species. This may explain the difference between the present study and the conclusions of molecular analysis methods. Furthermore, without quantization of gene mutation effects, using only formulas to describe the karyotype structure creates limitations in our ability to fully determine the genetic relationship. Ideally, genetic and karyological information should be combined in phylogenetic analyses.

In view of this, more detailed cephalopod chromosome information is urgently needed to facilitate comprehensive analyses of genetic relationships at the cytological level. Fluorescence in situ hybridization (FISH), which enables visualization of target DNA sites on chromosomes through a signal display using probes, has been widely applied in chromosomal localization (Colomba et al. 2002, Zhang et al. 2007, Wang et al. 2015, Escudero et al. 2016) and gene mapping (Ishizuka et al. 2016, Yasukochi et al. 2016) for many years; however, there is only one report of its use in cephalopods, which was based on the localization of telomere sequence (Adachi et al. 2014). In order to improve our understanding of cephalopod karyotypes, the development of chromosomal markers with higher resolution is needed to identify chromosome gene structure (Amar-Basulto et al. 2011). For example, if the complete telomere sequence positioning of *O. vulgaris* was available, we could determine whether chromosome translocation or rearrangements have taken place during its evolution.

In this study, we revealed the karyotypes of three octopods, bringing the total to nine reliable cephalopod karyotypes. Furthermore, this is the first study to determine the genetic relationship among these nine species at the cytological level by cluster analysis based on the karyotype evolutionary distance and resemblance-near coefficient. Our results demonstrated the feasibility of *D_e_* cluster analysis for cephalopod taxonomic classification, which could serve an important auxiliary means of routine phylogenetic analysis and provide insights into chromosome evolution.

## Supplementary materials

Supplementary material 1 Chromosome relative length, supplemental formulae Data type: statistical data Explanation note: Chromosome relative length, supplemental formulae and all of the original images are made available under the online digital repository Figshare, and it is free to access, in adherence to the principle of open data, more details in https://figshare.com/s/8d21a0db9ffe1f17d279 This dataset is made available under the Open Database License (http://opendatacommons.org/licenses/odbl/1.0/). The Open Database License (ODbL) is a license agreement intended to allow users to freely share, modify, and use this Dataset while maintaining this same freedom for others, provided that the original source and author(s) are credited.Jin-hai Wang, Xiao-dong Zheng
