# Supplementary material for: Comparison of the genetic relationship between nine Cephalopod species based on cluster analysis of karyotype evolutionary distance
Source: Comp Cytogenet. 2017 Jul 25;11(3):477–94. doi: 10.3897/CompCytogen.v11i3.12752 (PMC5646656; doi:10.3897/CompCytogen.v11i3.12752)
Supplement: Supplementary material 1 — Chromosome relative length, supplemental formulae [file comparative_cytogenetics-11-477-s001.docx]

The *D_e_* and *λ* are defined as follows:

$$D_{e}=-\ln\lambda$$

$$\lambda= \beta*\gamma$$

Where the *β* and *γ* are closeness coefficient and similarity coefficient, respectively. And they are calculated from the internal spur (*d_i_*) and external spur (*d_e_*) with:


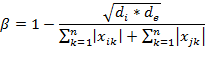

$$\gamma_{ij}=\frac{\sum_{k=1}^{n} x_{ik}*x_{jk}-\left( \sum_{k=1}^{n} x_{ik} \right)*\left( \sum_{k=1}^{n} x_{jk} \right)*\frac{1}{n}}{\sqrt{\sum_{k=1}^{n} \left( x_{ik}-\bar{x_{i}} \right)^{2}*\sum_{k=1}^{n} \left( x_{jk}-\bar{x_{j}} \right)^{2}}}$$

Where *i* (*j*) denotes the *i* (*j*)-th species. *k* denotes the *k*-th parameter, a total of *n* parameters. *x_ik_* and *x_jk_* denote the *k*-th parameter of *i*-th and *j*-th species.

Here, *d_i_* and *d_e_* were obtained in the following equations:


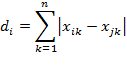

$$d_{e}=\left| \sum_{k=1}^{n} \left| x_{ik} \right|-\sum_{k=1}^{n} \left| x_{jk} \right| \right|$$
